# A study on the feasibility and initial outcomes of artificial intelligence-assisted preoperative planning for hip revision surgery

**DOI:** 10.3389/fsurg.2026.1690086

**Published:** 2026-03-11

**Authors:** Jiaqing Zhu, Tianwei Xia, Shanbin Zheng, Lu Wang, Dawei Huan, Xindong Yin, Yong Ma, Jirong Shen

**Affiliations:** 1 Department of Orthopaedics and Traumatology, Affiliated Hospital of Nanjing University of Chinese Medicine, Jiangsu Province Hospital of Chinese Medicine, Nanjing, China; 2 Department of General Surgery, Affiliated Hospital of Nanjing University of Chinese Medicine, Jiangsu Province Hospital of Chinese Medicine, Nanjing, China

**Keywords:** artificial intelligence, bone defects, preoperative planning, revision surgery, total hip arthroplasty

## Abstract

**Objective:**

The aim of this study was to explore the early and midterm efficacy of artificial intelligence (AI) involvement in assisted hip revision surgery and to summarize our clinical experience.

**Methods:**

Clinical data were collected from 38 patients (39 hips) who underwent hip revision surgery and preoperative planning using AI technology in our hospital between June 2019 and November 2024. The cohort included 17 men and 22 women, with a mean age of 70.33 ± 10.42 years (range 44–90 years). Mean follow-up time was 39.67 ± 16.76 months (range 7–72 months). The initial revision was performed in 37 hips, second revision in three hips, and third revision in one hip. The function of the affected limb was evaluated using Harris Hip Scoring System (HHS) at preoperative and postoperative nodes.

**Results:**

In this study, 38 patients were monitored for 7–72 months, with a mean follow-up of 34.64 ± 16.54 months. Matching of AI-planned prosthesis size with actual prosthesis size replaced during surgery: 3 cases (7.69%) showed a ±1 size discrepancy, 1 case (2.56%) showed a ±2 size discrepancy, and the remaining patients achieved precise matching. The HHS scores of all patients at 6 months postoperatively (82.99 ± 6.91) were higher than the preoperative scores (33.03 ± 7.36), and the difference was statistically significant (*P* < 0.05).

**Conclusion:**

AI technology makes complex revision surgeries simple by accurately formulating individual preoperative plans for hip revision surgery. The feasibility and preliminary efficacy of AI-assisted revision total hip arthroplasty surgery are satisfactory. AI-assisted complex orthopedic surgeries warrant further in-depth clinical research.

## 1 Introduction

Total hip arthroplasty (THA) is considered the ultimate treatment for conditions such as congenital hip dysplasia, ischemic necrosis of the femoral head, and osteoarthritis of the hip. Along with the increase in the number of THA cases, the number of patients requiring revision total hip arthroplasty (rTHA) has also increased annually (1). Previous studies have reported (2, 3) that the early causes of rTHA are primarily infection and aseptic loosening of the prosthesis, while later causes include osteolysis and aseptic loosening of the prosthesis. Compared with primary hip replacement surgery, revision surgery often involves more severe acetabular bone defects, which are more difficult to operate upon and have a higher failure rate. Mechanical abrasion of the bone bed by polyethylene, metal, or ceramic particles after THA surgery often results in periprosthetic osteolysis, causing loosening of the prosthesis. Removal of the prosthesis during revision surgery also tends to cause medically induced bone loss or fractures of the acetabulum and proximal femur, thus affecting the stability of the revision prosthesis. Therefore, the key to successful realization of hip revision surgery is to achieve the following three goals (4, 5): (1) effective management of the bone defect; (2) mechanical stability of the hip joint; and (3) restoration of the normal hip joint center position.

The joint replacement market is growing rapidly due to factors such as population aging and rising demands. However, there remains considerable room for improvement in joint surgery systems and tools. At present, most hospitals in China still adopt relatively outdated manual or 2D digital systems for preoperative planning, whereas leading hospital in the U.S. use 3D systems, such as Stryker's MAKO robot. Traditional preoperative planning of hip revision surgery is characterized by difficulties in assessing bone defects, imprecise determination of prosthetic loosening, and limited prosthesis selection. AI is expected to address these clinical challenges (6, 7). The AIHIP system first completes 3D image modeling using CT scans. Next, artificial intelligence—through an algorithm that gathers big data from the Chinese population and the doctor's professional experience—simulates multiple options before the surgery and selects the most suitable individualized, customized plan from among tens of thousands of models involving different permutations and combinations. Early studies have noted (8–10) that AI-assisted initial total hip arthroplasty has demonstrated high precision. Building on this, we retrospectively analyzed the patient data from 38 cases of rTHA performed at Jiangsu Provincial Hospital of Traditional Chinese Medicine between June 2019 and November 2024 (all of them used the AIHIP system for surgical planning prior to surgery). Our aim was to explore the clinical value of AI-assisted preoperative planning in hip revision surgery and to summarize our revision experience.

## 2 Materials and methods

### 2.1 Patient selection criteria

Inclusion criteria: (1) patients with acetabular bone defects of Paprosky type II and III requiring hip revision surgery (confirmed by two independent senior orthopedic surgeons based on standardized evaluation of preoperative hip X-rays and CTs); (2) patients who voluntarily accept AI-assisted revision surgery; and (3) patients with more than 6 months of follow-up and complete clinical data.

Exclusion criteria: (1) patients with contraindications to surgery or those unable to undergo surgery; (2) patients unwilling to participate in this study; and (3) patients with missing clinical data. Patient demographics are shown in Table 1.

**Table 1:** Clinical data of the patients.

General Information	The short-term group
Age (years)	70.33 ± 10.42
Gender (example)	*N* = 38
Male	17
Female	22
BMI (kg/m^2^)	24.24 ± 3.54
Side (example)	*N* = 39
Left	15
Right	24
Paprosky staging	*N* = 39
II A	14
II B	12
II C	7
III A	5
III B	1
Time from initial replacement to current renovation (years)	14.18 ± 6.20
Surgical time (min)	219.39 ± 96.34
Surgical approach (Example)	*N* = 39
Front	4
Back	35
Preoperative HHS (points)	33.03 ± 7.36
Hospitalization time (days)	14.72 ± 7.22
Postoperative time out of bed (days)	38.08 ± 42.66
Follow-up time (month)	39.67 ± 16.76
Which rTHA (example)	*N* = 39
First	35
Second	3
Third	1
Reason for revision	*N* = 39
Femoral stalk loose	8
Loose acetabular cup	4
Loose cup and femoral stem	3
Inner lining wear	3
Periprosthetic fracture	3
Dislocation	1
Infection	0

### 2.2 Population study

This study was designed as a retrospective case series. Between June 2019 and November 2024, a total of 38 patients (39 hips) from Jiangsu Provincial Hospital of Chinese Medicine met this selection criteria and were included in our study. There were 17 men and 22 women, with a mean age of 70.33 ± 10.42 years (range 44–90 years) and mean follow-up time of 39.67 ± 16.76 months (range 7–72 months). The initial revision involved 37 hips, three underwent second revision, and one underwent third revision.

### 2.3 AI-assisted preoperative planning

(1) Prior to the surgery, patients underwent pelvic frontal and lateral radiographs and CT examinations of the hip joint. CT adopted the new Fourier Convolution Block (FCB) to address the limitations of convolutional neural networks in capturing the global receptive field and to improve imaging speed. Hip CT encompassed the entire pelvis and extended 15 cm below the lesser trochanter of the femur.
(2) We need to convert the patient's CT scans from DICOM format to “.cmg” format for import into the AIHIP software. This software incorporates deep learning models based on neural networks—specifically segmentation models and 3D recognition models—which utilize U-Net as a baseline to precisely divide the skeleton into multiple specific bone segment regions. By integrating manually labeled bone-specific coordinate positioning points with an automatic search engine using big data learning, the most suitable prosthesis model for each patient is generated (11).

The AI-HIP system was utilized for preoperative planning. This system employs a deep convolutional neural network and is trained on a large multicenter dataset of hip joint CT scans from several top-tier hospitals in China to perform automatic 3D segmentation of the pelvis and detection of key anatomical landmarks. This model generates the initial surgical plan, including the recommended implant type, size, and position (cup tilt angle/version, stem forward tilt), as well as the predicted biomechanical results. Then, surgeons will review the AI-generated plan based on their clinical expertise and manually adjust it if necessary, finally determining the surgical plan. This process ensures that the final plan combines data-driven optimization with surgical judgment.

**Figure 1:**
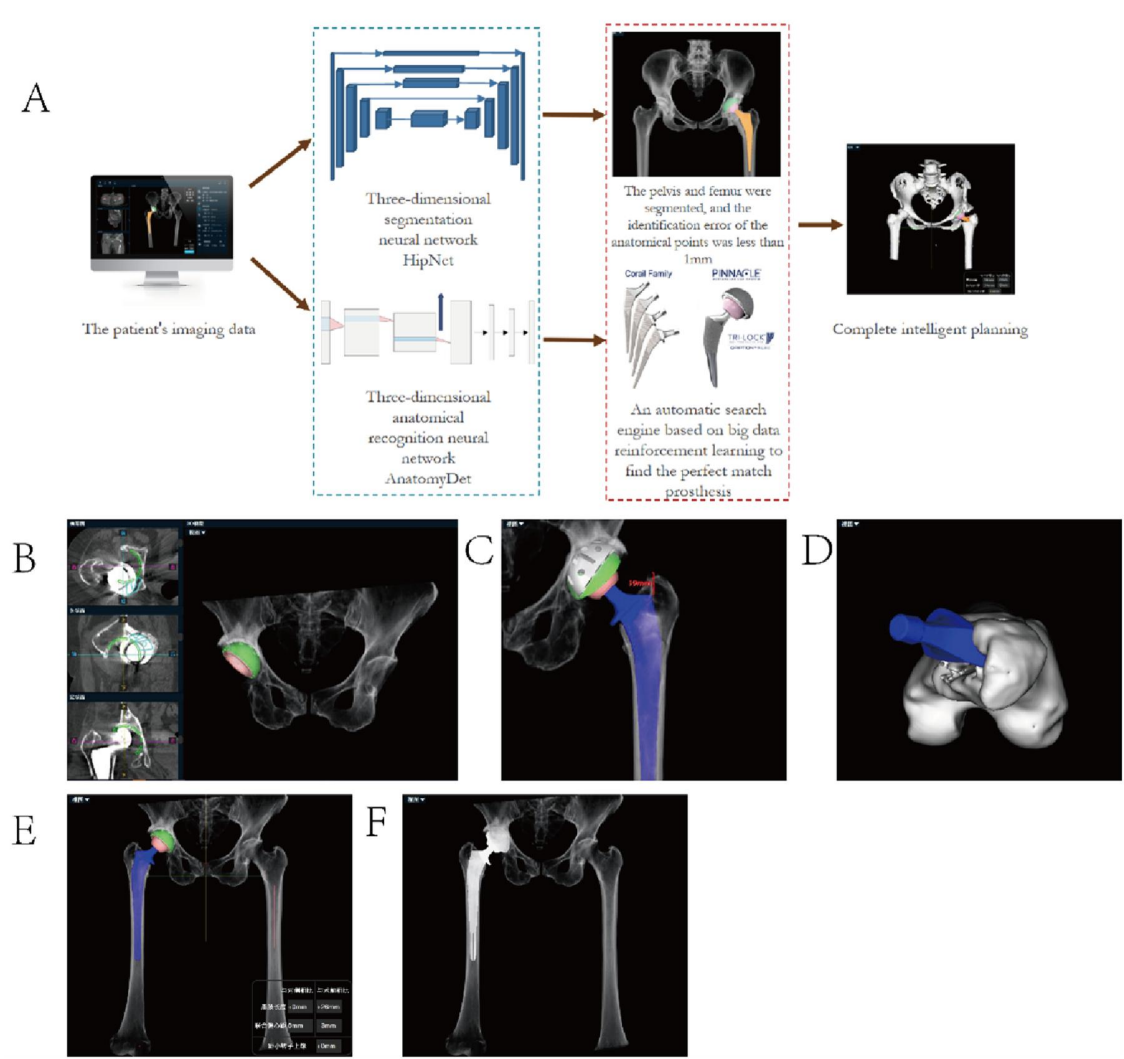
AIHIP system preoperative planning flowchart and typical examples. **(A)** Calculate the optimal placement of the socket cup; **(B)** calculate the placement of the femoral stem and measure the apical–shoulder distance; **(C)** plan the anterior tilt angle of the femur; **(D)** simulate the overall postoperative 3D view; and **(E)** simulate the anteroposterior X-ray film of the hip joint after the operation. **(F)** Post-operative CT scan of the hip joint in anterior-posterior position.

For details on the specific process, see Figure 1.

### 2.4 Surgical methods

The surgeries primarily adopted the posterolateral approach (for patients whose original incision was the anterior approach or Harding incision, a direct anterior incision was performed in the lateral position). Hyperplastic tissue around the joint and the dislocated hip joint was cleared, the loose prosthesis was removed, and a prosthesis of the appropriate size was implanted, based on the patient's specific condition in accordance with the AI preoperative plan. Based on our surgical experience, three distinct preoperative plans for rTHA were formulated, with the primary decision criterion being the severity of acetabular bone loss as classified by the Paprosky system (12):

Plan A: This strategy was designated for contained cavitary defects (Paprosky types I, IIA, IIB). The approach involved using a porous metal acetabular component (e.g., a trabecular metal cup or multihole cup) supplemented with morselized allograft bone as needed to address the contained deficit.
Plan B: This plan was intended for major segmental defects with compromised structural support (Paprosky types IIC, IIIA). The strategy employed a combination of a metal augment and a reinforcement ring [e.g., utilizing Cup-Cage or Cup-on-Cup techniques; by implanting two acetabular cups of different sizes, the inner cup (support cup) filled the area of the bone defect and provided initial stability, while the outer cup (true cup) was fixed to both the support cup and the remaining host bone by cementing or press-fit technique to form a composite structure that restored anatomical shape and mechanical stability to the acetabulum] to restore the acetabular column and rim, often in conjunction with bone grafting.
Plan C: This plan was reserved for the most severe cases with massive bone loss and pelvic discontinuity (Paprosky type IIIB, IIIC). The definitive solution involved implanting a custom 3D-printed acetabular prosthesis alongside significant bone grafting to achieve stability.

During the process of replacing the long handle on the femoral side, femoral fractures are prone to occur. In such cases, we typically used sternal steel wires, plates, or allogeneic bone plates for binding and fixation during the operation.

### 2.5 Perioperative management

For preoperative management, patients with underlying diseases (e.g., diabetes, hypertension) discontinued medication 1 day before surgery. Patients taking drugs such as reserpine or clopidogrel were required to discontinue for at least 2 weeks before surgery. Prior to surgery, ensure blood reserves are prepared according to the patient's condition, along with an adequate supply of various implant sizes and emergency equipment. Postoperatively, patients were encouraged to actively exercise, focusing on muscle contraction. After discharge, patients were referred to rehabilitation hospitals to gradually restore lower-limb function under the guidance of professional rehabilitation physicians.

### 2.6 Observational indicators of efficacy

Preoperative AI-generated designs were compared with intraoperative replacement prosthesis and the occurrence of complications. The function of the affected limb was evaluated before and after surgery using the HHS and other measures. The matching rate between the AI-generated preoperative plan and the actual replacement prosthesis was evaluated according to the following criteria (10): If the prosthesis model was the identical before and after surgery, it was considered an exact match; if there was a difference of ±1, it was considered a fair match; and if there was a difference of ±2 or more, it was considered a mismatch. In terms of cup implantation, based on the “Lewinneck safety zone” criterion (13), the position of the acetabular prosthesis was considered accurate if the anterior tilt angle was 15° ± 10° and the abduction angle was 40° ± 10°.

### 2.7 Statistical methods

Data were analyzed using SPSS 25.0 statistical software. We expressed the mean ± standard deviation for metric data that conform to a normal distribution. Count data were analyzed using ANOVA. Correlation analysis was performed using Bland–Altman plot or Cohen's kappa. A significance level of *α* = 0.05 was used.

## 3 Results

### 3.1 Surgical condition

A total of 39 hips (38 patients) were included in this study, of which 14 hips underwent replacement of the acetabular liner and femoral head, seven hips underwent replacement of the acetabular cup, liner, and artificial femoral head, five hips underwent replacement of the femoral stem and femoral head, and 13 hips underwent total revision of the acetabular and femoral stem prostheses. Perform the surgical procedure according to the plan outlined in Section 2.4: Among all patients, 36 underwent Plan A, including 2 cases with metal spacers; 2 underwent Plan B, including 1 case using cup-cage technique and 1 case using cup-on-cup technique; 1 underwent Plan C. Patients with proximal femoral fractures underwent plate and wire fixation in 6 cases and plate and screw fixation in 1 case. There were no missing data in this study, and complete datasets of both AI-assisted and traditional planning measurements were available for all cases.

### 3.2 Postoperative prosthesis survival status

The follow-up period for the 38 patients in this study ranged from 7 to 72 months, with an average follow-up time of 34.64 ± 16.54 months. Among the 39 hips, one hip developed thigh hematoma 5 months after surgery, and was treated with myotomy, drainage, myofasciotomy, and decompression surgery, combined with multiple postoperative blood transfusions (RH + type suspended oligoleukocytes), anti-inflammatory medicines (ceftriaxone sodium), and multiple medication changes. Half a month after the surgery, the wound healed well and test indexes were normal. Moreover, three hips experienced dislocation at 29, 31, and 55days postoperatively. The function of the affected limbs recovered after manipulation/incision and internal fixation at our hospital. No complications were observed in the remaining patients.

### 3.3 HHS situation

The patients' preoperative HHS was 33.03 ± 7.36. At 6 months postoperatively, the HHS was 82.99 ± 6.91, higher than the preoperative HHS, with a statistically significant difference (*P* < 0.05), as shown in Table 2.

**Table 2:** The patient suffered from HHS after the operation.

Stats	One week	Six months
*N* = 39	55.42 ± 8.51*	82.99 ± 6.91*^,^ **
*t*	12.430	15.713
*P*	<0.001	<0.001

* Indicates a comparison with the preoperative period, *P* < 0.05.

** Indicates a comparison with the last follow-up, *P* < 0.05.

### 3.4 Prosthesis prediction accuracy and correlation analysis

The position of the prosthesis was found to be within the safe zone in all the patients after surgery. In terms of AI-assisted preoperative planning of prosthesis type as matched with intraoperative replacement prosthesis, there was an error of ± 1 in three cases (7.69%), an error of ± 2 in one case (2.56%), and precise matching for the rest of the patients. The actual matching rate of the AI-assisted design was 92.31% on the acetabular side (statistically significant, *P* < 0.01) and 97.44% on the femoral side (statistically significant, *P* < 0.005), as shown in Table 3.

**Table 3:** Results of comparison between AIHIP planning prosthesis models and actual replacement prosthesis models.

Prosthetic site	Difference between preoperative design and actual (size)			Preoperative design and practical relevance	
	0	1	2	*k*	*P*
Acetabular side	36	2	1	0.466	<0.01
Femoral side	38	1	0	0.488	<0.005

### 3.5 Typical cases

#### 3.5.1 Case 1

A 69-year-old female patient had undergone right total hip arthroplasty for femoral head necrosis 10 years previously. She sought treatment for right hip pain aggravated by trauma for 1 month. Preoperative examination revealed a periprosthetic fracture of the femur, loosening of the hip prosthesis, severe osteoporosis, and a periprosthetic bone defect. The acetabular bone defect was classified as Paprosky type IIB. Preoperatively, AI-assisted planning was applied to measure the patient's hip CT and preview the surgery. We decided to use a 56-mm socket cup prosthesis to act as a bone defect filler, with a 48-mm socket cup placed for medial fixation, a 16.5-gauge uncemented femoral stem punched into the bone marrow cavity of the femur, and an in-built ball head (36-mm, +5) filler. The mesoprosthesis model was consistent with the preoperative plan.

**Figure 2:**
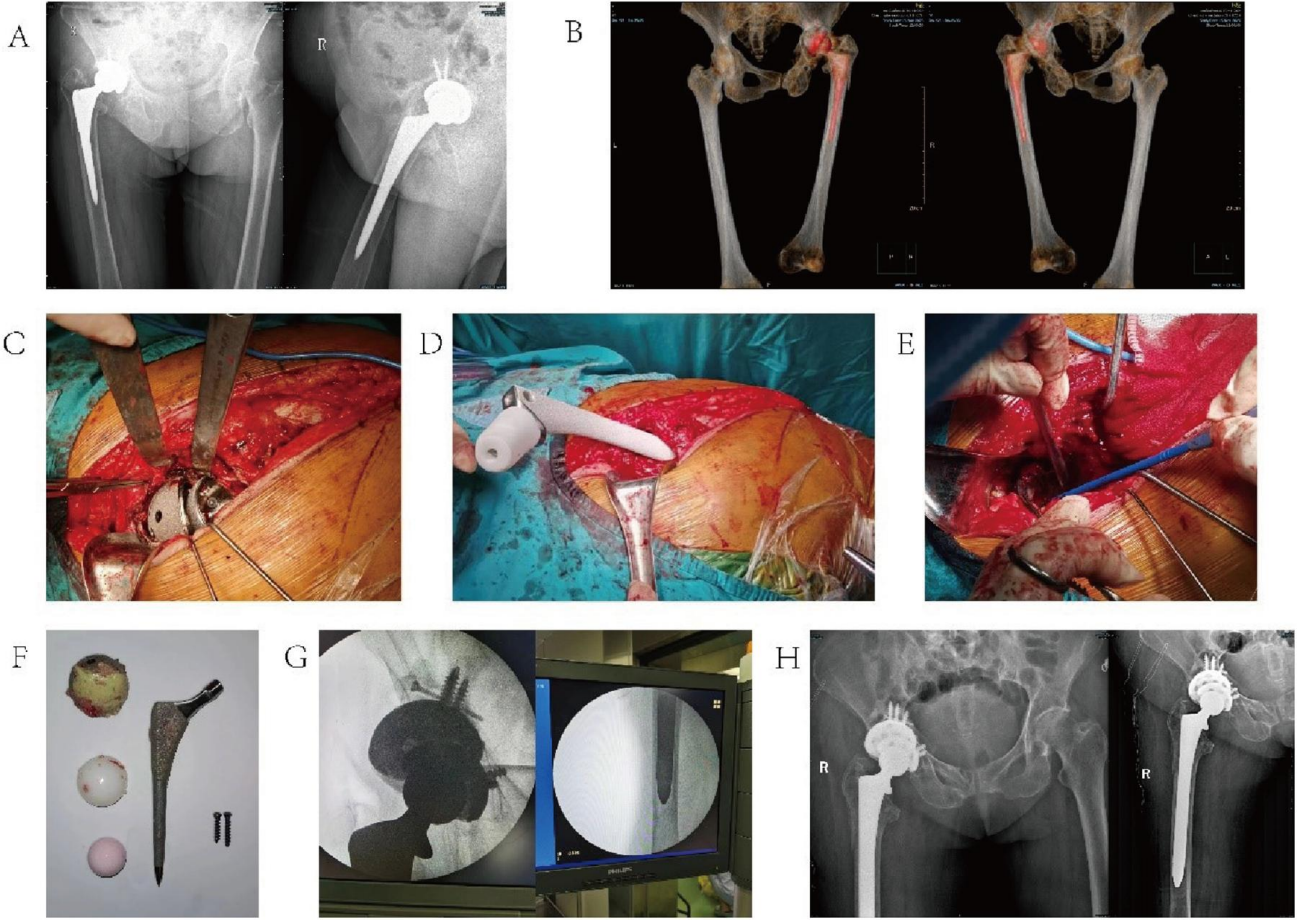
Typical case 1. **(A)** Preoperative hip X-ray; **(B)** preoperative CT; **(C)** intraoperative exposure of the acetabulum; **(D)** intraoperative placement of the bi-acetabular cup; **(E)** intraoperative installation of the femoral stem; **(F)** intraoperative removal of the prosthesis; **(G)** intraoperative fluoroscopy; and **(H)** postoperative hip X-ray.

For details, see Figure 2.

#### 3.5.2 Case 2

An 83-year-old male patient had undergone a left-side total hip arthroplasty for a femoral neck fracture 5 years earlier. He presented with pain in his left hip persisting for 1 year. The preoperative diagnosis was loosening of the hip prosthesis. The 52-mm acetabular cup was replaced with a 13.5-gauge femoral stem during operation.

**Figure 3:**
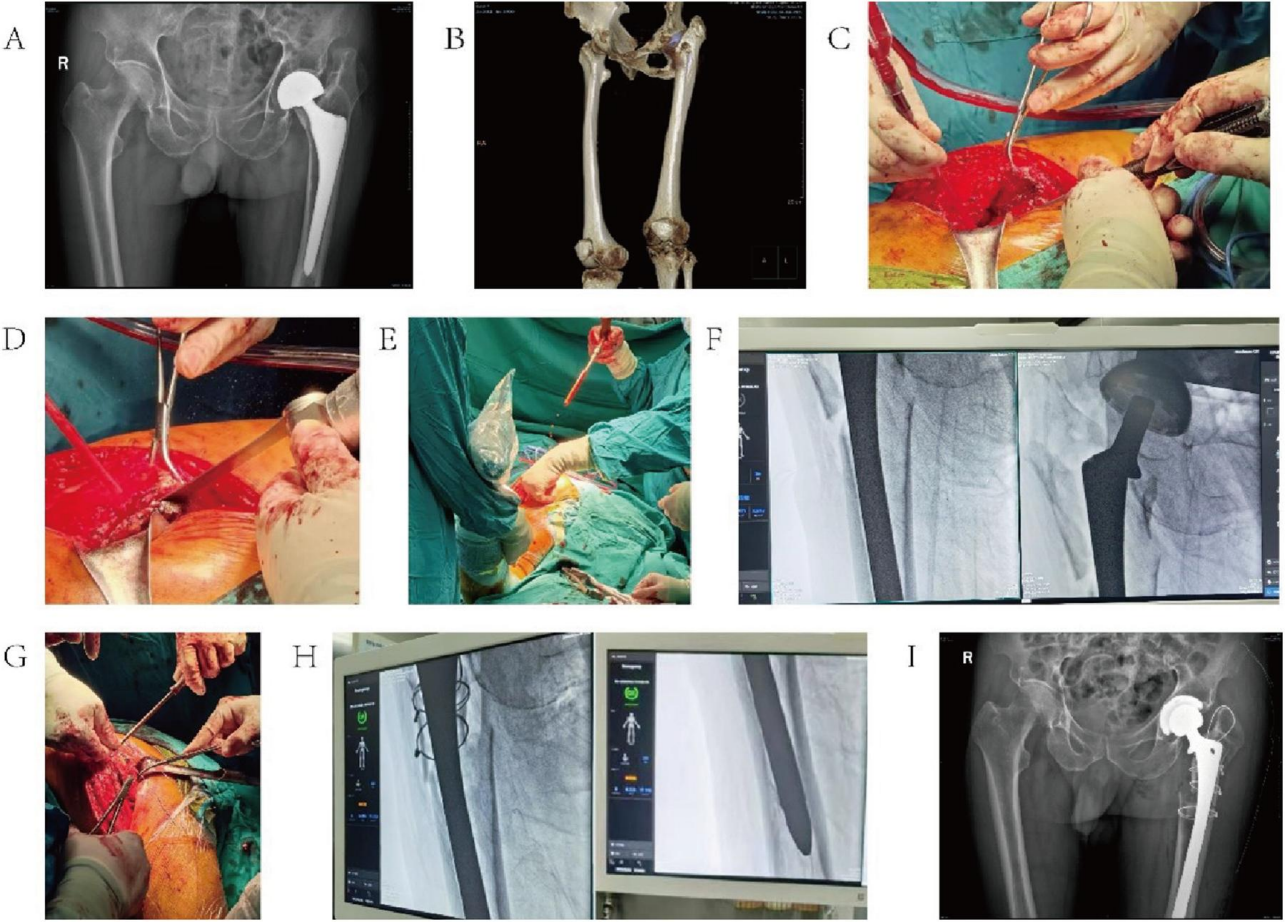
Typical case 2. **(A)** Preoperative hip X-ray; **(B)** preoperative CT; **(C)** scraper to clean the periprosthetic conjunctival tissue around the femur; **(D)** pendulum saw to remove the excess bone near the greater trochanter; **(E)** reamer to expand the medulla oblongata and open the sclerotic band of the distal femur; **(F)** intraoperative finding of proximal femur fracture; **(G)** bone grafting of the proximal femur and fixation with sternum wire ties; **(H)** intraoperative fluoroscopy; and **(I)** postoperative hip X-rays.

For details, see Figure 3.

## 4 Discussion

AI applications in clinical practice are playing an increasingly important role. For example, surgical simulators using AI assessment systems have enhanced our understanding of surgical expertise and have the potential to reduce skill heterogeneity by complementing competency-based training curricula (14–18). Current research on AI has focused primarily on radiology, oncology, and pathology, with fewer studies in surgery (particularly orthopedics) by physicians (19–23). In a survey of 13,806 participants from 43 countries on AI applications in healthcare, Busch et al. found that the majority of patients expressed support for the use of AI in healthcare, but also expressed concern regarding AI dominating diagnosis of diseases (24).

Acetabular side revision of artificial hip joints is a difficult operation because of the complex and variable anatomy and proximity to important organs and blood vessels. For surgeons, determining the appropriate size of the acetabular cup for revision surgery while simultaneously establishing the optimal position of the acetabular rotation center presents a significant challenge. The AI 3D planning system has unique advantages compared with traditional 3D planning. In particular, the AIHIP system employs a Transformer_unet mathematical algorithm with a deep learning model trained on a large revised case datasets, enabling not only 3D segmentation but also prediction of optimal implant size, position, and orientation by learning expert surgical patterns. This surpasses the manual, trial-and-error approach of standard software. Regarding patient selection criteria, we recommend that patients meeting the AIHIP resolution selection criteria be considered for AI-assisted preoperative planning in total hip arthroplasty. Agreement between AI preoperative planning and final intraoperative results was evaluated based on several continuous variables, including implant size, component position (e.g., 3D coordinates of the cup center), component orientation, and restoration of biomechanical parameters (e.g., leg length discrepancies, global offsets). Recognizing these metrics allows for a more comprehensive assessment of the strengths and weaknesses of AI planning.

Adequate preoperative planning can accurately predict prosthesis position and type, thus improving the surgical outcome. Inadequate preoperative preparation and failure to achieve a good match between the prosthesis and the bone during surgery can lead to gaps at the interface between the prosthesis and the bone, which in turn affects the stability of the prosthesis, and in the long term can lead to complications such as loosening of the prosthesis, pain, and reduced of arthroplasty viability (25, 26). With AI assistance, surgeons can preview and repeatedly simulate the surgical operation in front of the computer, transforming the joint replacement surgery into an open-book exam. Moreover, by fitting the acetabulum into a reasonable spherical mathematical model, AI can calculate the size of the cushion needed in revision surgery. In this manner, (1) the operation time can be shortened; (2) the trauma caused to the patient from repeated trial modeling during the operation can be reduced; (3) the use of unnecessary pads can be reduced, easing the economic burden on the patient; (4) the Harris score of the patient can be improved during late follow-up; and (5) the patient can realize early mobilization and deconditioning, conducive to accelerated recovery. Compared with traditional 2D and 3D preoperative planning, AI-assisted preoperative planning demonstrates higher accuracy (27–32). Huo et al. (8) reported an acetabular cup accuracy rate of 93.2% with AI-assisted planning for THA. Chinese scholars have similarly confirmed a high predictive accuracy of AI preoperative planning systems for assisted primary total hip replacement (11, 33).

However, there are relatively few studies on AI-based preoperative planning for complex initial or multiple revision hip surgeries. Unlike direct total hip replacement surgery, hip revision surgery may not always be straightforward to plan for optimal prosthesis fit outcomes. Patients with complex acetabular defects need a treatment plan combining AI-assisted preoperative simulation and the surgeon’s comprehensive understanding of the acetabular structure. The following are some available surgical options: allograft bone graft combined with acetabular cage, custom triflange acetabular component, cup-cage reconstruction technique, Jumbo cup combined with porous metal cushion, pelvic traction combined with porous tantalum cup, and porous tantalum cup combined with different shape-matched porous metal cushions. The final choice of surgery must remain a joint decision between the surgeon and the operative team. Hip revision surgery guided by preoperative planning improves consistency between planned and actual prosthesis positioning, which can significantly improve patients' postoperative Harris scores and demonstrates good clinical value (34). In this study, the AI preoperative planning system effectively reduced metal artifacts around the hip joint and accurately assessed hip defects. In all 38 patients (39 hips), the overall accuracy was 97.4% on the femoral side, higher than the acetabular side (92.3%). We attribute this to the visual error generated when the operator grinds the acetabular depth intraoperatively.

In cases of AI-assisted surgery, the observed hematoma incidence was 2.6% and the dislocation incidence was 7.7%, which aligns with the range reported for traditional repair methods. Chronic expanding hematoma (CEH) after THA is recognized as an infrequent but serious complication, though direct comparative rates are limited in the current literature (35). Dislocation rates in non-TM cup revisions (8,245 cases) demonstrate comparable variability, with meta-analyses highlighting implant selection as a key technical factor (36). Patient-specific risk factors—such as age ≥40–42 years, female sex, Tönnis angle ≥15°, and severe articular cartilage damage—are established predictors of poor outcomes (37). Notably, prior arthroscopy increases dislocation risk in THA patients (38), while under-resection during initial procedures remains a leading cause of revision (39). The AI planning system primarily contributed to defect quantification (addressing complex acetabular irregularities) and implant positioning guidance (improving accuracy over conventional methods). However, this study design cannot establish causality between AI planning and complication reduction, as confounding variables like native bone loss severity or soft tissue integrity likely exert greater influence. Future randomized controlled trials are necessary to determine whether AI planning significantly reduces complication risks compared with conventional techniques.

At present, we routinely perform direct anterior hip revision in the lateral position in patients whose initial primary incision is an anterolateral approach or Harding's incision and who have an intact posterior short external rotator group. For patients who have a posterior lateral approach for the initial replacement, who may have difficulty in removing the femoral stem, who have severe bone defects in the posterior wall or posterior column of the acetabulum, and who need to reestablish the stability of the pelvic ring during the operation, we perform hip revision surgery with a posterior lateral approach. Summarizing the experience of the anterior approach, the scar tissue around the hip joint should be completely loosened, and if necessary, the surrounding muscles (such as the short external rotators) may also be gradually loosened until the proximal end of the femur can be elevated and the Homann retractor can be inserted. Then, the affected limb can be completely exposed to the femur. In the face of complex acetabular bone defects, some scholars have proposed to increase the surgical field on the acetabular side by stripping the starting point of the broad fascia tensor muscle from the anterior superior skeletal spine (40). This can be actively tried with the assistance of intraoperative fluoroscopy. During the perioperative period, the prosthesis type of the patient's artificial hip joint is confirmed before surgery. The patient's original acetabular side ceramic liner, which is difficult to clamp, should be slowly loosened and removed by tapping. Blood bank preparations must also be completed prior to surgery. For patients suspected of having an infection, preparations for intraoperative blood testing should be maintained at all times. If necessary, representatives of the prosthesis company should be asked to provide sufficient prosthetic instruments to complete the operation. When encountering difficulty in extracting femoral prostheses, we recommend enlarging the medullary cavity at both the proximal and distal femur (using rigid and flexible drills) to facilitate removal. This procedure carries a risk of proximal femoral fracture. To ensure stability during prosthetic replacement, we employ a split fixation technique using bone plates on the contralateral ilium.

This study has some limitations: (1) It was a retrospective case series with a small sample size. Larger, multicenter prospective studies comparing AIHIP with traditional preoperative planning are needed in order to obtain more definitive results. (2) The current AIHIP system is primarily designed for preoperative planning of Depuy products. Cooperation with multiple prosthesis companies needs to be considered so as to provide more choices for physicians and patients to obtain the best clinical results. (3) The AIHIP system requires preoperative patient CT imaging data and incurs planning costs, resulting in an increase in the patient's expenses. However, in this study, the costs remained within an acceptable range. (4) The feasibility study lacked a control group undergoing traditional preoperative planning. Therefore, the findings should be interpreted as preliminary evidence of the technique's feasibility, with comparative efficacy requiring validation in future controlled studies.

This study demonstrates that AI-assisted preoperative planning is a feasible and accurate strategy for complex revision total hip arthroplasty. The technology achieved a high degree of concordance between the planned and implemented surgical steps, underscoring its practical utility in the operating room. Most importantly, the application of this approach was associated with significant clinical benefits, as evidenced by the dramatic and statistically significant improvement in Harris Hip Scores from 33.03 ± 7.36 preoperatively to 82.99 ± 6.91 at 6-month follow-up. The broader implication of our findings is that AI-assisted planning can serve as a powerful tool to enhance surgical precision and decision-making in challenging clinical domains. By providing a data-driven, three-dimensional blueprint, AI has the potential to reduce operative unpredictability and improve the standardization of care. For clinical practice, this technology promises to support surgeons, particularly those with less experience in complex revisions, in achieving optimal implant positioning and functional outcomes. For future research, our work sets the stage for comparative effectiveness studies—ideally, randomized controlled trials—to conclusively determine whether AI planning leads to superior long-term implant survival and lower complication rates compared with conventional planning. The integration of predictive analytics for patient-specific outcomes represents the next frontier for this technology.

## 5 Conclusion

Artificial intelligence simplifies complex revision surgery by accurately formulating individual preoperative plans for hip revision surgery. AI-assisted rTHA demonstrated surgical feasibility and preliminary efficacy, and the AIHIP planning technique merits clinical adoption. This study confirms the accuracy and predictability of AI in complex hip revision surgery, contributing to the advancement of digital and personalized orthopedic surgery.

## Data Availability

The original contributions presented in the study are included in the article/Supplementary Material, further inquiries can be directed to the corresponding authors.

## References

[1] Kurtz S Ong K Lau E Mowat F Halpern M. Projections of primary and revision hip and knee arthroplasty in the United States from 2005 to 2030. J Bone Joint Surg Am. (2007) 89(4):780–5. 10.2106/JBJS.F.00222 17403800

[2] Fryhofer GW Ramesh S Sheth NP. Acetabular reconstruction in revision total hip arthroplasty. J Clin Orthop Trauma. (2020) 11(1):22–8. 10.1016/j.jcot.2019.11.004 32001979 PMC6985018

[3] Goldman AH Sierra RJ Trousdale RT Lewallen DG Berry DJ Abdel MP. The Lawrence D. Dorr surgical techniques & technologies award: why are contemporary revision total hip arthroplasties failing? An analysis of 2500 cases. J Arthroplasty. (2019) 34(7S):S11–6. 10.1016/j.arth.2019.01.031 30765230

[4] Wessling M Gebert C Hakenes T Dudda M Hardes J Frieler S Reconstruction of Paprosky III defects with custom-made implants: do we get them in the correct position?: short-term radiological results. Bone Joint J. (2022) 104-B(10):1110–7. 10.1302/0301-620X.104B10.BJJ-2022-0508.R1 36177641

[5] Xiong C Meng D Ni R Cai H. Metal augments used in revision hip arthroplasty: a systematic review and single-arm meta-analysis. J Arthroplasty. (2023) 38(2):389–96.e1. 10.1016/j.arth.2022.08.010 35964855

[6] Zhu J Zheng S Sun J Ma B Zhang C Zhang C Efficacy of an artificial intelligence preoperative planning system for assisting in revision surgery after artificial total hip arthroplasty. BMC Surg. (2025) 25(1):58. 10.1186/s12893-024-02752-1 39920717 PMC11804043

[7] Geda MW Tang YM Lee CKM. Applications of artificial intelligence in orthopaedic surgery: a systematic review and meta-analysis. Eng Appl Artif Intell. (2024) 133:108326. 10.1016/j.engappai.2024.108326

[8] Huo J Huang G Han D Wang X Bu Y Chen Y Value of 3D preoperative planning for primary total hip arthroplasty based on artificial intelligence technology. J Orthop Surg Res. (2021) 16(1):156. 10.1186/s13018-021-02294-9 33627149 PMC7903792

[9] Zhang XL Wang KZ. The future of joint surgery: the application of digital orthopaedic technology in joint surgery. Chin J Orthop. (2021) 41(8):525–31. 10.3760/cma.j.cn121113-20210303-00214

[10] Wu D Chai W Liu XY An YC Zhang YL Chen JY Study on artificial intelligence-based algorithm for acetabular cup in total hip arthroplasty. Chin J Orthop. (2021) 41(3):176–85. 10.3760/cma.j.cn121113-20201110-00653

[11] Ding R Wang Q Liu Y Zhang QD Zhang NF Guo WS Application and accuracy analysis of artificial intelligence three-dimensional preoperative planning in total hip replacement. Chin J Orthop. (2022) 19(2):33–8. 10.3969/j.issn.1672-5972.2022.02.007

[12] Zhu JQ Sun JH Liu JZ Ma WB Zhang CY Zhang C Effectiveness analysis of revision surgery after total hip arthroplasty assisted by artificial intelligence preoperative planning system. Chin J Reparative Reconstr Surg. (2024) 38(04):455–60. 10.7507/1002-1892.202312099 PMC11024537 38632066

[13] Lewinnek GE Lewis JL Tarr R Compere CL Zimmerman JR. Dislocations after total hip-replacement arthroplasties. J Bone Joint Surg Am. (1978) 60(2):217–20. 10.2106/00004623-197860020-00014 641088

[14] Birkmeyer JD Finks JF O'Reilly A Oerline M Carlin AM Nunn AR Michigan Bariatric Surgery Collaborative. Surgical skill and complication rates after bariatric surgery. N Engl J Med. (2013) 369(15):1434–42. 10.1056/NEJMsa1300625 24106936

[15] Stulberg JJ Huang R Kreutzer L Ban K Champagne BJ Steele SR Association between surgeon technical skills and patient outcomes. JAMA Surg. (2020) 155(10):960–8. 10.1001/jamasurg.2020.3007. Erratum in: JAMA Surg. 2020 155(10):1002. doi: 10.1001/jamasurg.2020.4676. Erratum in: JAMA Surg. 2021 156(7):694. doi: 10.1001/jamasurg.2021.1953. 32838425 PMC7439214

[16] Fazlollahi AM Bakhaidar M Alsayegh A Yilmaz R Winkler-Schwartz A Mirchi N Effect of artificial intelligence tutoring vs expert instruction on learning simulated surgical skills among medical students: a randomized clinical trial. JAMA Netw Open. (2022) 5(2):e2149008. 10.1001/jamanetworkopen.2021.49008 35191972 PMC8864513

[17] Feldman MJ Hoffer EP Conley JJ Chang J Chung JA Jernigan MC Dedicated AI expert system vs generative AI with large language model for clinical diagnoses. JAMA Netw Open. (2025) 8(5):e2512994. 10.1001/jamanetworkopen.2025.12994 40440012 PMC12123466

[18] Lex JR Di Michele J Koucheki R Pincus D Whyne C Ravi B. Artificial intelligence for hip fracture detection and outcome prediction: a systematic review and meta-analysis. JAMA Netw Open. (2023) 6(3):e233391. 10.1001/jamanetworkopen.2023.3391 36930153 PMC10024206

[19] Vasey B Ursprung S Beddoe B Taylor EH Marlow N Bilbro N Association of clinician diagnostic performance with machine learning-based decision support systems: a systematic review. JAMA Netw Open. (2021) 4(3):e211276. 10.1001/jamanetworkopen.2021.1276 33704476 PMC7953308

[20] Liu X Faes L Kale AU Wagner SK Fu DJ Bruynseels A A comparison of deep learning performance against health-care professionals in detecting diseases from medical imaging: a systematic review and meta-analysis. Lancet Digit Health. (2019) 1(6):e271–97. 10.1016/S2589-7500(19)30123-2. Erratum in: Lancet Digit Health. 2019 1(7):e334. doi: 10.1016/S2589-7500(19)30160-8. 33323251

[21] Hosny A Parmar C Quackenbush J Schwartz LH Aerts HJWL. Artificial intelligence in radiology. Nat Rev Cancer. (2018) 18(8):500–10. 10.1038/s41568-018-0016-5 29777175 PMC6268174

[22] Kapoor R Walters SP Al-Aswad LA. The current state of artificial intelligence in ophthalmology. Surv Ophthalmol. (2019) 64(2):233–40. 10.1016/j.survophthal.2018.09.002 30248307

[23] Liu H Ding N Li X Chen Y Sun H Huang Y Artificial intelligence and radiologist burnout. JAMA Netw Open. (2024) 7(11):e2448714. 10.1001/jamanetworkopen.2024.48714 39576636 PMC11584928

[24] Busch F Hoffmann L Xu L Zhang LJ Hu B García-Juárez I Multinational attitudes toward AI in health care and diagnostics among hospital patients. JAMA Netw Open. (2025) 8(6):e2514452. 10.1001/jamanetworkopen.2025.14452 40493367 PMC12152705

[25] Barrow JA Divecha HM Panchani S Boden R Porter ML Board TN. Does oversizing an uncemented cup increase post-operative pain in primary total hip arthroplasty? Eur J Orthop Surg Traumatol. (2019) 29(1):97–102. 10.1007/s00590-018-2240-9 29855788

[26] Townsend S Kim SE Pozzi A. Effect of stem sizing and position on short-term complications with canine press fit cementless total hip arthroplasty. Vet Surg. (2017) 46(6):803–11. 10.1111/vsu.12666 28460422

[27] Archibeck MJ Cummins T Tripuraneni KR Carothers JT Murray-Krezan C Hattab M Inaccuracies in the use of magnification markers in digital hip radiographs. Clin Orthop Relat Res. (2016) 474(8):1812–7. 10.1007/s11999-016-4704-8 26797909 PMC4925406

[28] Weber M Woerner ML Springorum HR Hapfelmeier A Grifka J Renkawitz TF. Plain radiographs fail to reflect femoral offset in total hip arthroplasty. J Arthroplasty. (2014) 29(8):1661–5. 10.1016/j.arth.2014.03.023 24857334

[29] Tsai TY Dimitriou D Li G Kwon YM. Does total hip arthroplasty restore native hip anatomy? Three-dimensional reconstruction analysis. Int Orthop. (2014) 38(8):1577–83. 10.1007/s00264-014-2401-3 24966079 PMC4115107

[30] Wu L Zhao X Lu ZD Yang Y Ma L Li P. Accuracy analysis of artificial intelligence-assisted three-dimensional preoperative planning in total hip replacement. Jt Dis Relat Surg. (2023) 34(3):537–47. 10.52312/jdrs.2023.1059 37750257 PMC10546848

[31] Liu Y Zhang Z Wang W Yu C Liu C Huang Z Artificial intelligence planning and 3D printing augmented modules in the treatment of a complicated hip joint revision: a case report. Front Surg. (2023) 10:1237075. 10.3389/fsurg.2023.1237075 37795146 PMC10546305

[32] Chen X Li S Wang Y Liu X Zhang Y Qiu G Artificially intelligent three-dimensionally-printed patient-specific instrument improves total hip arthroplasty accuracy. J Arthroplasty. (2023) 38(10):2060–7.e1. 10.1016/j.arth.2022.12.017 36535443

[33] Wu D Liu XY Zhang YL Chen JY Tang PF Chai W. Research and application of artificial intelligence based three-dimensional preoperative planning system for total hip arthroplasty. Chin J Reparative Reconstr Surg. (2020) 34(09):1077–84. 10.7507/1002-1892.202005007 PMC8171718 32929897

[34] Hao Y Luo D Wu J Wang L Xie K Yan M A novel revision system for complex pelvic defects utilizing 3D-printed custom prosthesis. J Orthop Translat. (2021) 31:102–9. 10.1016/j.jot.2021.09.006 34976730 PMC8683605

[35] Wu C Wang H Yu T Bao Q Wen J Zhang J Chronic expanding hematomas arising over a decade post primary total hip arthroplasty. J Orthop Translat. (2025) 54:199–213. 10.1016/j.jot.2025.03.017 41064584 PMC12502923

[36] Shen X Qin Y Li Y Tang X Xiao J. Trabecular metal versus non-trabecular metal acetabular components for acetabular revision surgery: a systematic review and meta-analysis. Int J Surg. (2022) 100:106597. 10.1016/j.ijsu.2022.106597 35288338

[37] Kraeutler MJ Terle PM Malempati M Dhillon J Samuelsson K Mei-Dan O. Risk factors for failure of hip arthroscopy in patients with borderline dysplasia include a Tönnis angle ≥15°, age ≥40 to 42 years, female sex, anterior wall index <0.35, labral debridement, and preexisting hip osteoarthritis: a systematic review. Arthroscopy. (2025) 41(7):2636–45. 10.1016/j.arthro.2024.10.021 39490543

[38] Liu Q Tian Z Pian K Duan H Wang Q Zhang H The influence of prior arthroscopy on outcomes of primary total lower extremity arthroplasty: a systematic review and meta-analysis. Int J Surg. (2022) 98:106218. 10.1016/j.ijsu.2021.106218 34995806

[39] Schoof LH Hartwell MJ. Editorial commentary: navigating the Cam: balancing precision, cost, and patient-reported outcomes for computer-assisted femoral resections. Arthroscopy. (2025) 41(12):5164–6. 10.1016/j.arthro.2025.08.026 40962078

[40] Li ZL Shen KW Xiao Y Lin FT Weng Y Feng EY Clinical evaluation of direct anterior approach in revision total hip arthroplasty. Chin J Bone Joint Surg. (2022) 15(4):262–8. 10.3969/j.issn.2095-9958.2022.04.04

